# Supplementary material for: Non-A Blood Type Is a Risk Factor for Poor Cardio-Cerebrovascular Outcomes in Patients Undergoing Dialysis
Source: Biomedicines. 2023 Feb 16;11(2):592. doi: 10.3390/biomedicines11020592 (PMC9953354; doi:10.3390/biomedicines11020592)
Supplement: Supplementary file 1 [file biomedicines-11-00592-s001.zip › biomedicines-2211334-supplementary/Table S1.pdf]

Table S1. The correlation between dialytic interval and echocardiographic parameters

	**R**	**p value**
LV ejection fraction, %	-0.06	0.28
LV diastolic diameter, mm	0.21	< 0.001
LV systolic diameter, mm	0.16	0.003
LV wall thickness, mm	-0.05	0.34
LV mass index, g/m^2	-0.01	0.91
Left arterial diameter, mm	0.16	0.003
E/E'	-0.02	0.78

Abbreviations; LV, left ventricular; E/E', ratio of the early diastolic transmitral flow velocity to mitral annular velocity.
