# Supplementary material for: Non-A Blood Type Is a Risk Factor for Poor Cardio-Cerebrovascular Outcomes in Patients Undergoing Dialysis
Source: Biomedicines. 2023 Feb 16;11(2):592. doi: 10.3390/biomedicines11020592 (PMC9953354; doi:10.3390/biomedicines11020592)
Supplement: Supplementary file 1 [file biomedicines-11-00592-s001.zip › biomedicines-2211334-supplementary/Table S2.pdf]

Table S2. Cox proportional hazard analyses including dialytic date-interval

**Characteristic**	**Multivariable analyses**		
	**HR**	**95% CI**	**P value**
Blood type			
Type A, vs. non-A type	0.46	0.26-0.81	0.007
Basic data			
Age, per 10-year increase	1.48	1.18-1.85	0.001
Primary disease of dialysis			
Diabetes mellitus, vs. non-diabetes mellitus	1.24	0.76-2.02	0.39
History of cardio- or cerebrovascular disease	1.14	0.63-2.07	0.66
Medication			
Anti-platelet or anti-coagulation	1.92	1.07-3.41	0.028
Echocardiography			
LVEF, per 10-% increase	0.78	0.63-0.96	0.021
LV mass index, per 10-g/m^2 increase	1.07	1.01-1.13	0.021
Dialytic date-interval, per 1-day increase	1.02	0.79-1.31	0.90

Abbreviations; LV, left ventricular; E/E', ratio of the early diastolic transmitral flow velocity to mitral annular velocity.
