# Supplementary material for: Non-A Blood Type Is a Risk Factor for Poor Cardio-Cerebrovascular Outcomes in Patients Undergoing Dialysis
Source: Biomedicines. 2023 Feb 16;11(2):592. doi: 10.3390/biomedicines11020592 (PMC9953354; doi:10.3390/biomedicines11020592)
Supplement: Supplementary file 1 [file biomedicines-11-00592-s001.zip › biomedicines-2211334-supplementary/Table S3.pdf]

Table S3. The incidence of each clinical outcome in each blood type

ABO blood type	A	B	O	AB	Total
The number of patients, n	149	81	99	36	365
Heart failure, n (%)	3 (2%)	6 (7%)	13 (13%)	2 (6%)	24
Ischemic heart event, n (%)	6 (4%)	4 (5%)	5 (5%)	1 (3%)	16
Cerebrovascular event, n (%)	5 (3%)	4 (5%)	7 (7%)	6 (17%)	22
Sudden death, n (%)	2 (1%)	7 (9%)	6 (6%)	0 (0%)	15
All cause death, n (%)	19 (13%)	18 (22%)	20 (20%)	5 (14%)	62
