# Supplementary material for: Non-A Blood Type Is a Risk Factor for Poor Cardio-Cerebrovascular Outcomes in Patients Undergoing Dialysis
Source: Biomedicines. 2023 Feb 16;11(2):592. doi: 10.3390/biomedicines11020592 (PMC9953354; doi:10.3390/biomedicines11020592)
Supplement: Supplementary file 1 [file biomedicines-11-00592-s001.zip › biomedicines-2211334-supplementary/Table S4.pdf]

Table S4. The incidence of each clinical outcome in patients with A or AB and patients with B or O

**ABO blood type**	**A or AB**	**B or O**	P value
The number of patients, n	185	180	
Heart failure, n (%)	5 (3%)	19 (11%)	0.002
Ischemic heart event, n (%)	7 (4%)	9 (5%)	0.57
Cerebrovascular event, n (%)	11 (6%)	11 (6%)	0.95
Sudden death, n (%)	2 (1%)	13 (7%)	0.003
All cause death, n (%)	24 (13%)	38 (21%)	0.038
