# Supplementary material for: Non-A Blood Type Is a Risk Factor for Poor Cardio-Cerebrovascular Outcomes in Patients Undergoing Dialysis
Source: Biomedicines. 2023 Feb 16;11(2):592. doi: 10.3390/biomedicines11020592 (PMC9953354; doi:10.3390/biomedicines11020592)
Supplement: Supplementary file 1 [file biomedicines-11-00592-s001.zip › biomedicines-2211334-supplementary/Table S5A.pdf]

Table S5A. The detail causes of death

**ABO blood type**	**A**	**B**	**O**	**AB**	**Total**
The number of patients, n	149	81	99	36	365
All cause death, n	19 (13%)	18 (22%)	20 (20%)	5 (14%)	62
Sudden death, n (%)	2	7	6	0	15
Infection, n (%)	8	3	6	2	19
Heart failure, n (%)	1	3	3	0	7
Acute coronary syndrome, n (%)	1	0	0	0	1
Cerebrovascular events, n (%)	0	1	1	1	3
Cancer, n (%)	2	0	1	2	5
The others, n (%)	5	4	3	0	12
