# Supplementary material for: Non-A Blood Type Is a Risk Factor for Poor Cardio-Cerebrovascular Outcomes in Patients Undergoing Dialysis
Source: Biomedicines. 2023 Feb 16;11(2):592. doi: 10.3390/biomedicines11020592 (PMC9953354; doi:10.3390/biomedicines11020592)
Supplement: Supplementary file 1 [file biomedicines-11-00592-s001.zip › biomedicines-2211334-supplementary/Table S5B.pdf]

Table S5B. The detail causes of death

ABO blood type	Type A	Non-A type	P value
The number of patients, n	149	216	
All cause death, n	19 (13%)	43 (19%)	
Sudden death, n (%)	2 (1%)	13 (6%)	0.027
Infection, n (%)	8 (5%)	11 (5%)	0.91
Heart failure, n (%)	1 (1%)	6 (3%)	0.15
Acute coronary syndrome, n (%)	1 (1%)	0 (0%)	0.23
Cerebrovascular events, n (%)	0 (0%)	3 (2%)	0.15
Cancer, n (%)	2 (1%)	3 (1%)	0.97
The others, n (%)	5 (3%)	7 (3%)	0.95
