# Supplementary material for: Non-A Blood Type Is a Risk Factor for Poor Cardio-Cerebrovascular Outcomes in Patients Undergoing Dialysis
Source: Biomedicines. 2023 Feb 16;11(2):592. doi: 10.3390/biomedicines11020592 (PMC9953354; doi:10.3390/biomedicines11020592)
Supplement: Supplementary file 1 [file biomedicines-11-00592-s001.zip › biomedicines-2211334-supplementary/Table S6.pdf]

Table S6. Cox proportional hazard analyses including history of atrial fibrillation

Characteristic	Multivariable analyses		
	HR	95% CI	P value
Blood type			
Type A, vs. non-A type	0.46	0.26-0.82	0.008
Basic data			
Age, per 10-year increase	1.47	1.17-1.83	0.001
Primary disease of dialysis			
Diabetes mellitus, vs. non-diabetes mellitus	1.24	0.76-2.03	0.38
History of cardio- or cerebrovascular disease	1.17	0.65-2.11	0.61
Medication			
Anti-platelet or anti-coagulation	1.86	1.04-3.33	0.038
Echocardiography			
LVEF, per 10-% increase	0.79	0.64-0.97	0.024
LV mass index, per 10-g/m^2 increase	1.07	1.01-1.13	0.022
History of atrial fibrillation	1.25	0.59-2.67	0.56

Abbreviations; LV, left ventricular; E/E', ratio of the early diastolic transmitral flow velocity to mitral annular velocity.
