# Supplementary material for: Non-A Blood Type Is a Risk Factor for Poor Cardio-Cerebrovascular Outcomes in Patients Undergoing Dialysis
Source: Biomedicines. 2023 Feb 16;11(2):592. doi: 10.3390/biomedicines11020592 (PMC9953354; doi:10.3390/biomedicines11020592)
Supplement: Supplementary file 1 [file biomedicines-11-00592-s001.zip › biomedicines-2211334-supplementary/Table S7.pdf]

Table S7. Cox proportional hazard analyses including valvular heart disease ≥ moderate

Characteristic	Multivariable analyses		
	HR	95% CI	P value
Blood type			
Type A, vs. non-A type	0.47	0.27-0.83	0.009
Basic data			
Age, per 10-year increase	1.42	1.13-1.78	0.003
Primary disease of dialysis			
Diabetes mellitus, vs. non-diabetes mellitus	1.23	0.75-2.01	0.41
History of cardio- or cerebrovascular disease	1.09	0.60-1.99	0.77
Medication			
Anti-platelet or anti-coagulation	1.93	1.08-3.45	0.027
Echocardiography			
LVEF, per 10-% increase	0.79	0.64-0.97	0.025
LV mass index, per 10-g/m^2 increase	1.06	1.00-1.12	0.042
Valvular disease ≥ moderate	1.53	0.85-2.75	0.15

Abbreviations; LV, left ventricular; E/E', ratio of the early diastolic transmitral flow velocity to mitral annular velocity.
